# Value of Whole-Thyroid CT-Based Radiomics in Predicting Benign and Malignant Thyroid Nodules

**DOI:** 10.3389/fonc.2022.828259

**Published:** 2022-05-05

**Authors:** Han Xu, Ximing Wang, Chaoqun Guan, Ru Tan, Qing Yang, Qi Zhang, Aie Liu, Qingwei Liu

**Affiliations:** ^1^ Department of Radiology, Shandong Provincial Hospital, Cheeloo College of Medicine, Shandong University, Jinan, China; ^2^ Department of Radiology, Shandong Provincial Hospital Affiliated to Shandong First Medical University, Jinan, China; ^3^ Department of Mammary Nail Surgery, Shandong Provincial Hospital Affiliated to Shandong First Medical University, Jinan, China; ^4^ Department of Research Center, Shanghai United Imaging Intelligence Inc., Shanghai, China

**Keywords:** thyroid disease, tomography, radiomics, diagnosis, differentiation

## Abstract

The objective of this research is to explore the value of whole-thyroid CT-based radiomics in predicting benign (noncancerous) and malignant thyroid nodules. The imaging and clinical data of 161 patients with thyroid nodules that were confirmed by pathology were retrospectively analyzed. The entire thyroid regions of interest (ROIs) were manually sketched for all 161 cases. After extracting CT radiomic features, the patients were divided into a training group (128 cases) and a test group (33 cases) according to the 4:1 ratio with stratified random sampling (fivefold cross validation). All the data were normalized by the maximum absolute value and screened using selection operator regression analysis and K best. The data generation model was trained by logistic regression. The effectiveness of the model in differentiating between benign and malignant thyroid nodules was validated by a receiver operating characteristic (ROC) curve. After data grouping, eigenvalue screening, and data training, the logistic regression model with the maximum absolute value normalized was constructed. For the training group, the area under the ROC curve (AUC) was 94.4% (95% confidence interval: 0.941–0.977); the sensitivity and specificity were 89.7% and 86.7%, respectively; and the diagnostic accuracy was 87.6%. For the test group, the AUC was 94.2% (95% confidence interval: 0.881–0.999); the sensitivity and specificity were 89.4% and 86.8%, respectively; and the diagnostic accuracy was 87.6%. The CT radiomic model of the entire thyroid gland is highly efficient in differentiating between benign and malignant thyroid nodules.

## Introduction

In recent years, thyroid nodules have become common diseases. The detection rate in the general population is approximately 65%. Most nodules are benign; only 5%–15% of nodules are malignant (1). The key factors in the diagnosis and treatment of thyroid nodules are to distinguish benign nodules from malignant ones, and to avoid overtreatment. Because CT examination can help evaluate the relationships between thyroid nodules and deep cervical structure, the metastasis of deep cervical lymph nodes, that can improve the accuracy of preoperative tumor staging, so CT examination is more and more used in thyroid nodules before operation. Radiomics improves the accuracy of predicting benign and malignant tumors by extracting high-throughput information from medical images (2). Most existing thyroid radiomic studies are based on single thyroid nodules, but many thyroid diseases appear multiple nodules that are irregular in shape and unclear in boundary, therefore, it is difficult to completely and accurately delineate thyroid lesion. Also, a single nodule cannot reflect the entire picture of thyroid lesions. Thus, our study delineates all thyroid tissues, and benign and malignant thyroid nodules were well predicted based on the CT radiomic characteristics of the entire thyroid gland, that can provide a noninvasive, accurate, and feasible diagnostic method for clinic flow.

## Information and Methods

### General Information

From March 2019 to July 2021, a total of 161 patients in the Provincial Hospital affiliated with Shandong First Medical University were retrospectively analyzed, including 53 benign cases and 108 malignant cases. The patients,42 males and 119 females, were aged 15–79 years with an average age of (47.8 years ± 13.8 years). All thyroid nodules were confirmed by surgery and pathology. The inclusion criteria were as follows: (1) no history of thyroid surgery,(2) no history of cervical radiotherapy, and (3) thyroid gland clearly intact upon CT imaging. Exclusion criteria included (1) unqualified histological examination,(2) unclear diagnosis, and (3) unavailable CT image.

### Instruments and Methods

All thyroid nodule subjects were scanned using a Philips Ingenuity 64 Spiral CT or a Siemens Force Dual Source CT. The patients were scanned while lying down, and the thyroid gland was well exposed. The CT scanning range was from the skull base to the thoracic entrance. Some patients had large thyroid gland volume, and the scope was appropriately expanded to include all thyroid tissues. The scanning parameters were as follows:tube voltage of 120kV, tube current of 80 mA, layer thickness of 3 mm, and layer spacing of 3 mm. The contrast agent, iohexol (purchased from Beijing Beilu Pharmaceutical, China), was intravenously injected at a dose of 1.5 mL/kg and an injection rate of 3.5 mL/s. The arterial phase images were collected 35 seconds after injection, and the venous phase images were collected 65 seconds after injection.

### Radiomic Feature Extraction

Three-phase CT images of the thyroid glands of all patients were uploaded to the uAI Scientific Research Portal (United Imaging Intelligence, China) in DICOM format. To reach an agreement through consultation, two neck radiologists, one with five years of diagnostic experience and one with ten years of diagnostic experience, used the software to sketch all thyroid tissue of each patient. The software automatically extracts 2600 radiomics features from the painted ROI, including 104 original features extracted from the original image and additional features extracted after 14 different filtering transformations. The original features include 18 gray statistics features,14 shape features, and 72 texture features. The 14 filters include box mean, additive gaussian noise, binomial blur image, curvatureflow, boxsigmaim-age, normalize, Laplacian sharpening, discrete gaussia, mean, speckle noise, recursive gaussian, shot noise, log (4 log-sigma:0.5 mm,1.0 mm,1.5 mm, and 2 mm), and wavelet (8 species: wavelet-LLL, wavelet-LLH, wavelet-LHL, wavelet-LHH, wavelet-HLL, wavelet-HLH, wavelet-HHL, and wavelet-HHH). We selected fivefold cross-validation for data grouping and randomly divided all data into five groups. One group was randomly selected as the test group, and the remaining four groups were training groups: all five groups of data were cycled. All data were normalized by absolute value maximum normalization, and the 16 eigenvalue subsets with the best performance were finally obtained by least absolute shrinkage and selection operator regression (LASSO) and K-best serial screening eigenvalue. In addition, the prediction model was established by logistic regression (LR) classifier.

**Figure 1:**
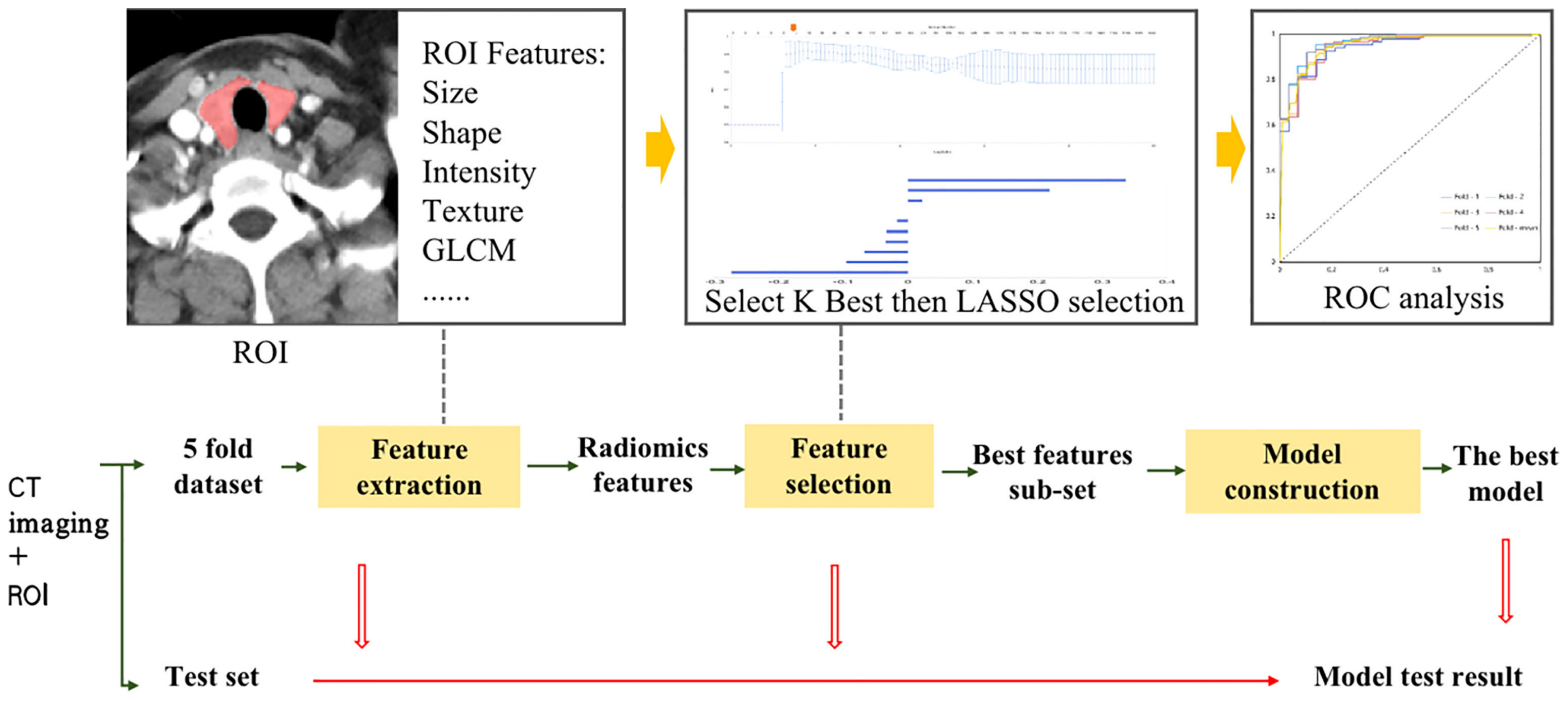
Flowchart of Radiomics Analysis.

See **Figure 1** for the specific flow.

### Statistical Processing

The sensitivity, specificity, accuracy, F1-score, and area under the ROC curve (AUC) of the predictive model in differentiating between benign and malignant thyroid nodules between the training group and the test group were evaluated by ROC curve of the subjects’ operating characteristics. A group was randomly selected to compare the distribution of benign malignancy and gender between the training group and the test group by χ^2^, and the age difference, which was statistically significant (*P* <.05), was calculated using an independent sample t-test (in accordance with normal distribution).

## Results

### General Information

A total of 161 patients were included in this study. There were 53 cases of benign nodules, including 40 cases nodular goiter; four cases of eosinophilic adenomas; three cases of Hashimoto’s thyroiditis; two cases of diffuse goiter; and four cases of follicular adenoma. There were 108 malignant cases:53 cases of papillary carcinoma, 50 cases of micropapillary carcinoma, three cases of follicular carcinoma, and two cases of medullary carcinoma. The thyroid tissue of all patients was completely delineated on three-phase CT scan images, and three ROIs were delineated in each patient, including benign ROIs (159) and malignant ROIs (324). A total of 2600 features were extracted from 483 ROIs;and 16 optimal feature subsets, including five morphological features, seven texture features, and four high-order features, were screened using absolute value maximum normalization preprocessing, K-best, and a selection operator regression analysis classifier.

**Figure 2:**
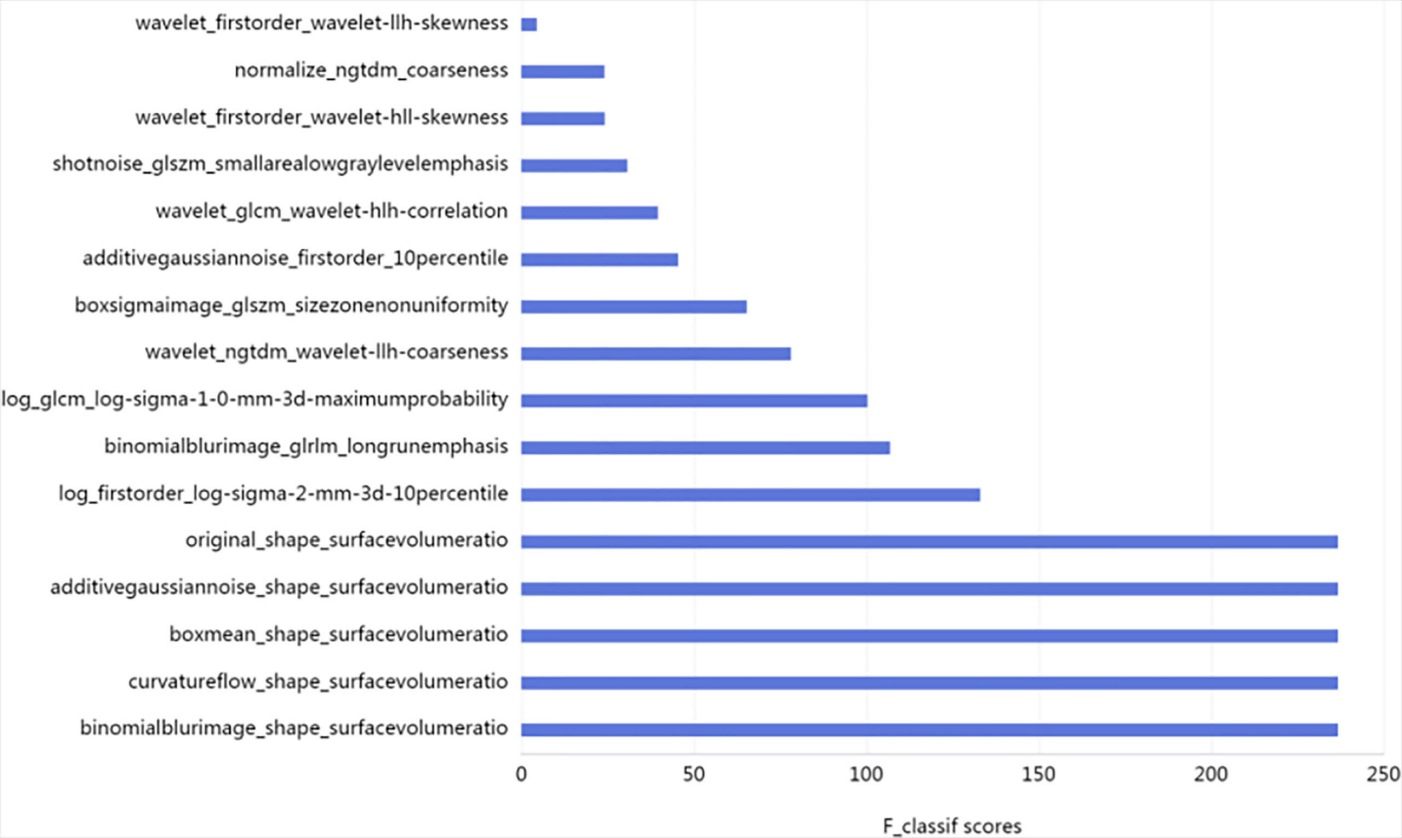
F1-Score of 16 Optimal Features (the higher the F1-score, the greater the feature weight).

The weights of each feature are shown in **Figure 2**.

### Radiomic Diagnostic Model

**Figure 3:**
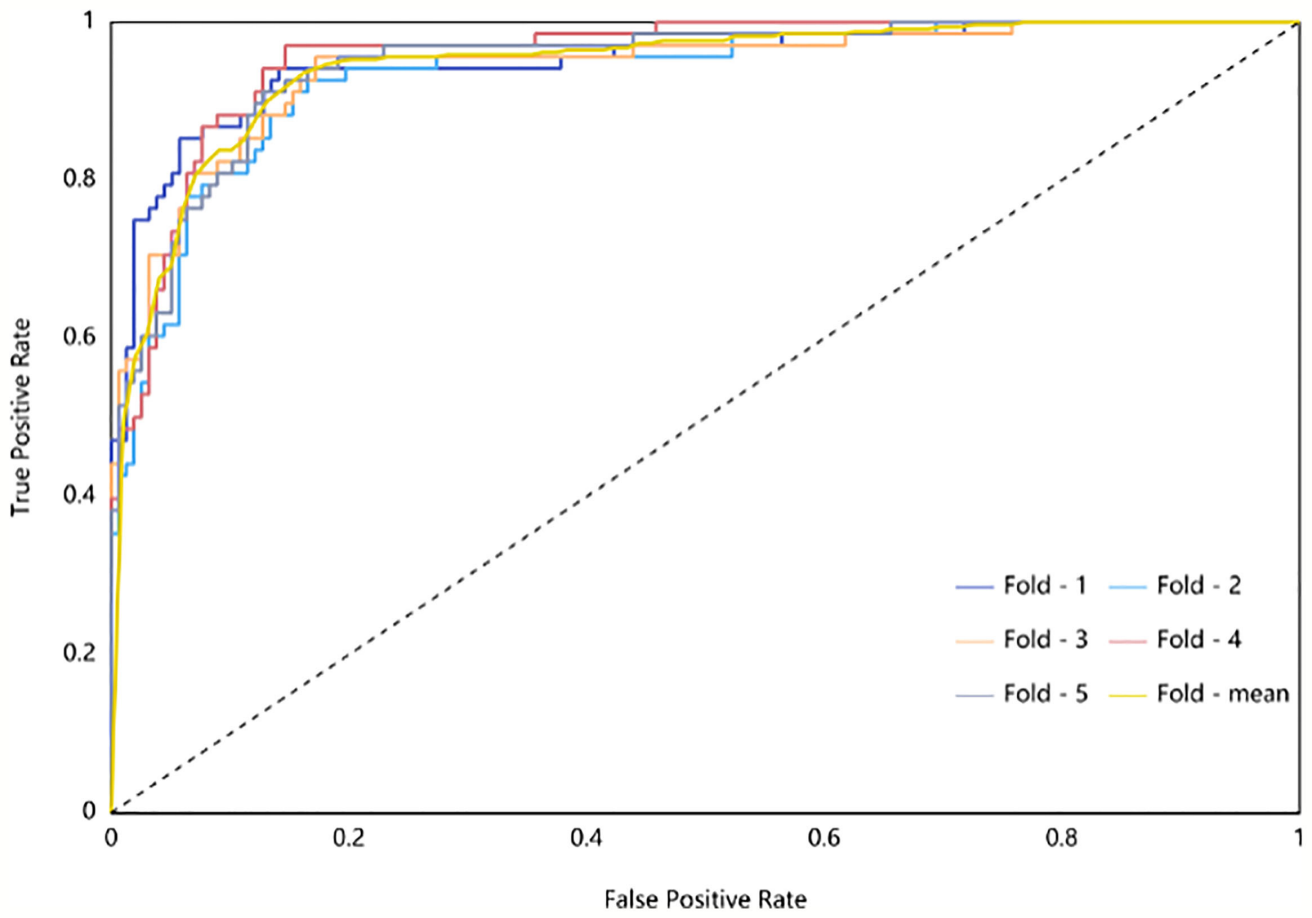
ROC Curve of Training Group (different subsets are shown by different-colored curves; the yellow curve is the mean value curve).

**Figure 4:**
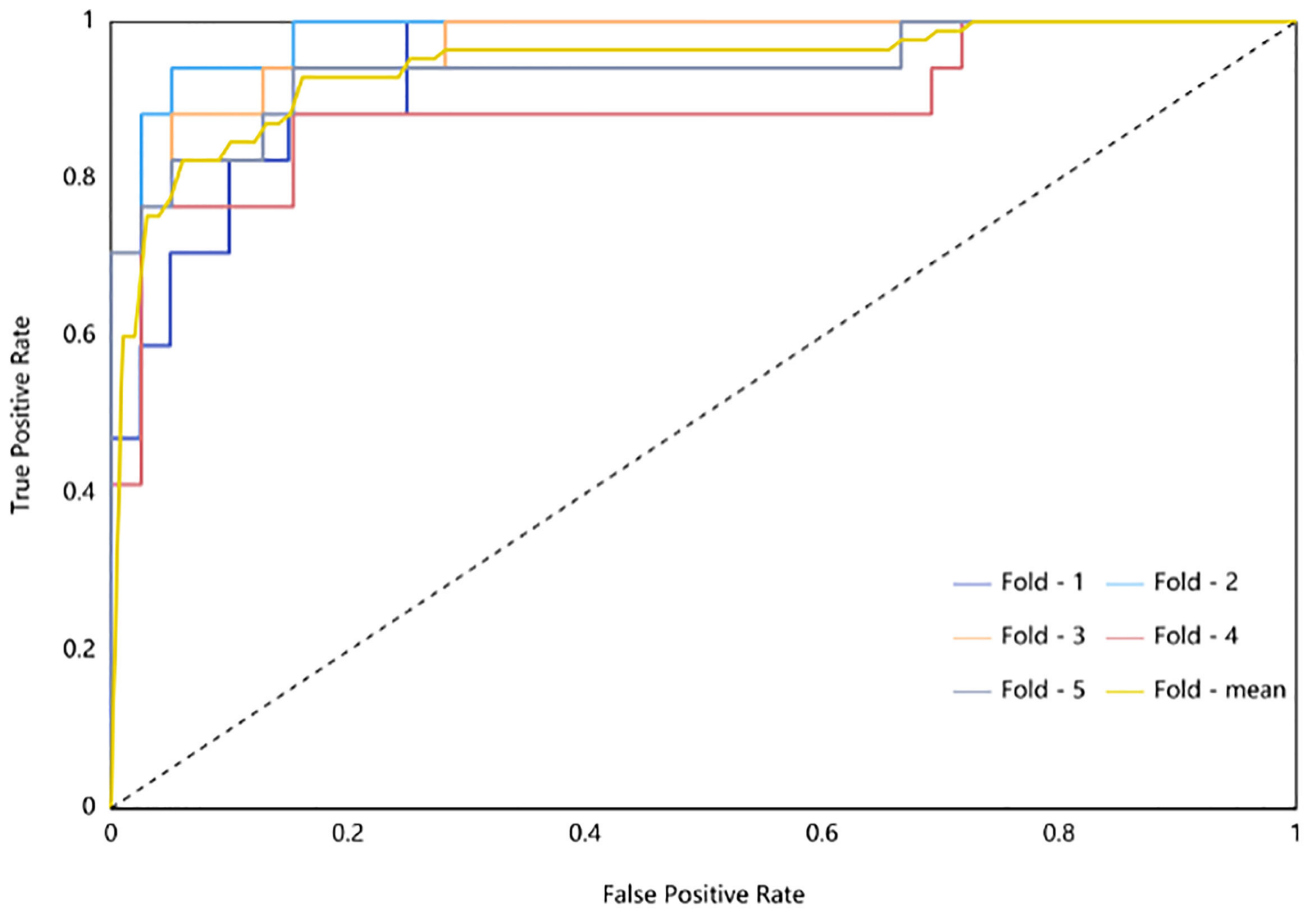
ROC Curve of Testing Group (different subsets are shown by different-colored curves; the yellow curve is the mean value curve).

The radiomic model of the maximum absolute value normalized-logistic regression was built. In the training group, the AUC value of the ROC curve was approximately0.944 (95% confidence interval: 0.941–0.977) show in **Figure 3**, the F1-score was 0.815, the sensitivity was 89.7%, the specificity was 86.7%, and the diagnostic accuracy was 87.6%. In the test group, the AUC was 0.942 (95% confidence interval: 0.881–0.999) show in **Figure 4**, the F1-score was 0.819, the sensitivity was 89.4%, the specificity was 86.8%, and the diagnostic accuracy was 87.6%.

## Discussion

Ultrasonography has been the optimal examination of the thyroid gland, but ultrasound examination relies excessively on the clinical experience of ultrasound doctors and has blind spots for deep neck structures (3). Thyroid CTs have advantages over ultrasound in these areas, rendering them helpful for preoperative staging and optimization of surgical plans. Thyroid CT scanning has been used as a supplementary examination, limited to low sensitivity in diagnosis. However, the emergence of radiomics has broken the ice; radiomics can mine a large amount of deep data from images and improve the accuracy of image-based diagnoses (2). Abundant research has been conducted on the use of this technology in the gastrointestinal tract, pulmonary nodules, breast tumors, and in other locations (4–6). In contrast, there is relatively little research on thyroid radiomics (7–10) and even less focused on CT images. Recent studies by Kong Dan et al. showed that the thyroid CT radiomics model has high diagnostic efficiency for differentiating between benign and malignant thyroid nodules (11). The AUCs of both the training group and the test group were greater than 90%, and the diagnostic accuracy was greater than 80%, which is close to the results of this study (the AUC of the training group was 94.4%, and the AUC of the test group was 94.2%). Hu Xiaoli et al.’s research is based on plain CT scanning of the thyroid gland to distinguish between benign and malignant thyroid nodules of less than 2 cm (12). The results showed that the diagnostic efficiency of this model remained satisfactory despite being based on plain CT scans, and the AUC values of both the training group and the test group were greater than 80%. All the above studies were conducted on single thyroid nodules. However, many thyroid diseases manifest multiple nodules with different morphologies and densities, so the study of single nodules cannot reflect whole lesion situations. Furthermore, some thyroid cancers demonstrate diffuse growth involving one lobe or the entire thyroid gland, which makes the thyroid gland diffuse swelling. When no obvious tumor nodules are found, it is difficult to delineate the boundary of the lesion (13, 14), which renders delineation of thyroid nodules difficult. This study involved sketching all the thyroid tissue, which can provide a complete extraction of both the overall data of thyroid lesions and the data surrounding these lesions. Compared with the single nodule study, the features extracted in this study were richer and more complete; and the operation was simpler and easier. The results show that the whole thyroid CT radiomics model has higher diagnostic efficiency and is stronger than the single nodule thyroid CT model.

In this study,16 subsets of optimal radiomic features were selected, including five morphological features, seven texture features, and four high-order features. The analysis of the weight ratio indicated that the maximum weight in this study was the morphological characteristics (surface–volume ratio), which is consistent with the single nodule study. These results suggest that the morphological features of the lesions are important factors in differentiating between benign and malignant lesions. Although we have not delineated the tumor itself, most malignant tumors are invasive, large, and unclear in boundary; and these characteristics affect the entire thyroid’s morphology, that differ from benign lesions.

Aside from morphological features, texture features were the most numerous features, including gray-level run-length matrix, gray-level co-occurrence matrix, neighborhood gray-level difference matrix, and gray-level size zone matrix. Texture features indicate the relative differences among different voxels and reflect the internal heterogeneity of tumors. The information revealed by texture features is usually invisible to the naked eye and is not affected by subjective factors. Malignant tumors are prone to necrosis and degeneration because of the formation of neovascularization in tumors, and the relative differences among voxels in malignant tumors are large. Benign tumors grow slowly and are relatively internally homogeneous; the relative differences among their voxels are small (2). The weight of a single texture feature is low, and a combination of multiple texture features greatly influences differentiation between benign and malignant thyroid nodules.

Although this study has achieved satisfactory results, it had shortcomings. First, the amount of data resulting from the study is relatively small, and the research focused on single-center data; therefore, there is a need to expand the multi-center sample size. Second, because there were few patients with lymph node metastasis in malignant cases, the distribution of data was biased, and the prediction of lymph node metastasis was incomplete. The next step is to continue collecting cases for the prediction of lymph node metastasis to develop a more comprehensive evaluation method for clinical practice. Third, in our department, we perform three phases scan at Thyroid CT enhancement examination. overlooking radiation burden, that need to further develop optimal scan sequences. Fourth, A uni-radiomic study was performed on CT images in this study, at the next step we should perform multi-radiomic study fusing with clinical information.

## Data Availability Statement

The raw data supporting the conclusions of this article will be made available by the authors, without undue reservation.

## Ethics Statement

Ethical review and approval was not required for the study on human participants in accordance with the local legislation and institutional requirements. The patients/participants provided their written informed consent to participate in this study.

## Author Contributions

XW and CG designed the study. HX, XW, CG, RT, QY, QZ, and AL collected and analyzed the data. HX interpreted the data and wrote the paper. All authors have read and approved the manuscript.

## Conflict of Interest

Author AL was employed by Shanghai United Imaging Intelligence Inc.

The remaining authors declare that the research was conducted in the absence of any commercial or financial relationships that could be construed as a potential conflict of interest.

## Publisher’s Note

All claims expressed in this article are solely those of the authors and do not necessarily represent those of their affiliated organizations, or those of the publisher, the editors and the reviewers. Any product that may be evaluated in this article, or claim that may be made by its manufacturer, is not guaranteed or endorsed by the publisher.
